# CTAB-Assisted Fabrication of Bi_2_WO_6_ Thin Nanoplates with High Adsorption and Enhanced Visible Light-Driven Photocatalytic Performance

**DOI:** 10.3390/molecules22050859

**Published:** 2017-05-22

**Authors:** Yuxue Zhou, Pengfei Lv, Xiangdong Meng, Yanping Tang, Pingping Huang, Xiaobing Chen, Xiaoshuang Shen, Xianghua Zeng

**Affiliations:** School of Physical Science & Technology, Yangzhou University, Yangzhou 225002, China; yxzhou@yzu.edu.cn (Y.Z.); flyinglyu@163.com (P.L.); mengxd@yzu.edu.cn (X.M.); typ1995@icloud.com (Y.T.); hpp494251869@163.com (P.H.); xbchen@yzu.edu.cn (X.C.)

**Keywords:** Bi_2_WO_6_ nanoplates, semiconductor photocatalyst, visible light-driven

## Abstract

Two-dimensional thin Bi_2_WO_6_ nanoplates have been fabricated using a cetyltrimethylammonium bromide (CTAB)-assisted hydrothermal method. We investigated the proposed formation mechanism based on the crystalline structures of the thin Bi_2_WO_6_ nanoplates. The high adsorption ability and excellent visible-light driven photocatalytic activities of the Bi_2_WO_6_ nanoplates were illustrated, in view of exposed (001) facets of nanoplates possessing faster separation of photo-generated charge carriers and increased catalytically active sites. Such a cost-effective way to obtain Bi_2_WO_6_ nanoplates offers new possibilities for the design of adsorptive semiconductor photocatalysts with strengthened photocatalytic activities.

## 1. Introduction

The semiconductor photocatalysis technique is reported to be an efficient and environmentally friendly method for the treatment of organic environment pollutants [1]. Due to the fact visible light accounts for 43% of sunlight compared to 4% from UV light, visible light responsive semiconductor photocatalysts have become a major focus of water treatment research in order to realize the full use of solar power and conserve energy [2]. As one of the simplest members of the aurivillius oxide family of layered perovskites, bismuth tungstate (Bi_2_WO_6_), with its narrow bandgap (ca. 2.6–2.7 eV) has aroused significant attention in view of its good photocatalytic performance in the degradation of chloroform, acetaldehyde and organic dyes under visible light irradiation [3,4,5,6,7]. Considering the close dependence of the photocatalytic performance of Bi_2_WO_6_ species on their morphology, Bi_2_WO_6_ photocatalysts of various sizes and shapes have been widely studied (such as Bi_2_WO_6_ nanoparticles [8], hierarchical Bi_2_WO_6_ microspheres [9], self-assembled microdiscs [10], etc.). Particularly, increasing attention has been recently focused on the two-dimensional Bi_2_WO_6_ micro- and nanostructures represented by mono-layered nanosheets [11] and ultrathin nanoplates [12], in view of their increased surface-to-volume ratio, faster separation of photogenerated electrons and holes with plenty of catalytically active sites and excellent structural stability [13], which are attractive and important features for improving the photocatalytic activities of Bi_2_WO_6_ under visible light irradiation.

Herein, two-dimensional thin Bi_2_WO_6_ nanoplates with round edges were firstly obtained using a simple hydrothermal reaction system employing Bi(NO_3_)_3_·5H_2_O and Na_2_WO_4_·2H_2_O as raw materials and cetyltrimethylammonium bromide (CTAB) as surfactant. High adsorption and enhanced visible light-driven photocatalytic performance of these Bi_2_WO_6_ thin nanoplates for the decomposition of rhodamine B (RhB) aqueous solution was demonstrated in detail.

## 2. Materials and Methods

### 2.1. Synthesis

Bi(NO_3_)_3_·5H_2_O, Na_2_WO_4_·2H_2_O and CTAB chemical reagents used in this work were of analytical grade, bought from Sinopharm Chemical Reagent Company (Shanghai, China) and were used without further purification. In a typical procedure for the synthesis of thin Bi_2_WO_6_ nanoplates, 2 mmol Bi(NO_3_)_3_·5H_2_O, 0.5 g CTAB, and 1 mmol Na_2_WO_4_#xB7;2H_2_O were respectively dissolved in 10 mL distilled water. Firstly, 10 mL CTAB aqueous solution was added dropwise to 10 mL Bi(NO_3_)_3_·5H_2_O aqueous solution and stirred for 30 min. Then 10 mL Na_2_WO_4_·2H_2_O aqueous solution was added to the above solution and the mixture solution was stirred for 1 h at room temperature until a precursor suspension was obtained. Finally, the resulting solution was transferred to a 50 mL Teflon-line autoclave, and maintained at 180 °C for 20 h. After the autoclave cooled down to room temperature naturally, the prepared samples were collected by centrifugation, washed with distilled water and absolute ethanol for several times, and then dried at 80 °C in a drying oven for 6 h. Commercial nano-TiO_2_ with size of 5–10 nm and phenol were purchased from Shanghai Aladdin Biochem Technology Company (Shanghai, China).

### 2.2. Characterization

The obtained products were characterized by X-ray diffraction (XRD, Philips X’ Pert Pro Super, Amsterdam, The Netherlands), field emission scanning electron microscopy (FESEM, Hitachi S-4800, Tokyo, Japan), transmission electron microscope (TEM, Phillips Tecnai-12, Amsterdam, The Netherlands), high-resolution transmission electron microscopy (HRTEM, FEI Tecnai G2 F30 S-TWIN, Hillsboro, OR, USA) and energy-dispersive X-ray (EDX) analysis was obtained with an EDAX detector installed on the same HRTEM. UV-visible diffuse reflectance spectra (DRS) were recorded on a UV-vis diffuse reflectance spectrum (Varian Cary 5000, Palo Alto, CA, USA). The Brunauer-Emmett-Teller (BET) surface area was measured using a Micrometrics ASAP 2020 (Micrometrics, Norcross, GA, USA).

### 2.3. Photocatalytic Activity Test

The photocatalytic activities of the Bi_2_WO_6_ samples were evaluated by the degradation of rhodamine B (RhB) or phenol under a simulated sunlight irradiation using a 300 W Xe lamp with a 420 nm cut-off filter as a light source. In each experiment, 50 mg of photocatalyst was added into 100 mL RhB of solution (1 × 10^−5^ mol/L) or phenol solution (15 mg/L). Prior to visible light illumination, the suspensions were magnetically stirred in the dark for 60 min to reach adsorption-desorption equilibrium between thin Bi_2_WO_6_ nanoplates and RhB aqueous solution, after that the solution was exposed to visible light irradiation with non-stop stirring. During visible light irradiation, about 4 mL of suspension was removed from the reactor at certain time intervals and centrifuged to get stop the deposition on the Bi_2_WO_6_ nanoplates photocatalyst. The filtrates were analyzed by recording changes of the absorption band maximum (553 nm) in the UV-vis spectra of RhB by using a spectrophotometer (UV-2700, Shimadzu, Suzhou, China). The photocatalytic efficiency was calculated according to the following equation:

$$\mathrm{Removal}\;\mathrm{efficiency}\;(\%)=\frac{C_{0}-C}{C_{0}}\times 100\%$$

where C_0_ and C represent the original concentration of RhB and phenol in dark, and the reaction concentration of RhB, phenol after a certain time visible-light irradiation, separately.

## 3. Results and Discussion

### 3.1. Structure and Morphology

**Figure 1:**
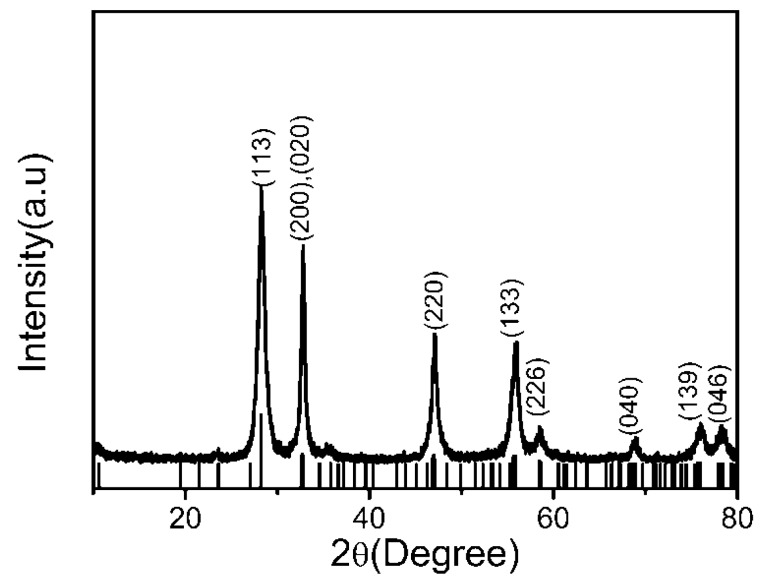
XRD pattern of Bi_2_WO_6_ thin nanoplates prepared at 180 °C for 20 h.

Two-dimensional Bi_2_WO_6_ thin nanoplates was firstly obtained by a CTAB-assisted hydrothermal method under 180 °C for 20 h. The XRD pattern of the Bi_2_WO_6_ thin nanoplate product is shown in Figure 1 where eight major sharp reflection peaks located at 2θ = 28.21°, 32.80°, 47.06°, 56.05°, 58.62°, 68.84°, 76.24°, 78.50° showed the well-crystallized orthorhombic phase of Bi_2_WO_6_ with lattice parameters of a = 5.457 Å, b = 5.436 Å, and c = 16.427 Å, which matches very well with the standard values (Powder Diffraction File No: 73-1126). No peaks of any other phases or impurities were detected in this pattern. The intensity ratio of the (200) or (020) peak to the (113) peak is 0.80, evidently higher than the standard value of 0.185, while the full width at half maximum (FWHM) of the (200) or (020) Bragg peak is narrower than that of the (113) peak and indicates a higher grain size of Bi_2_WO_6_ along the (100) and (010) directions compared to the (001) direction. Therefore both the above analysis indicate that Bi_2_WO_6_ nanoplates with special anisotropic features will grow along the (001) plane [12,14].

**Figure 2:**
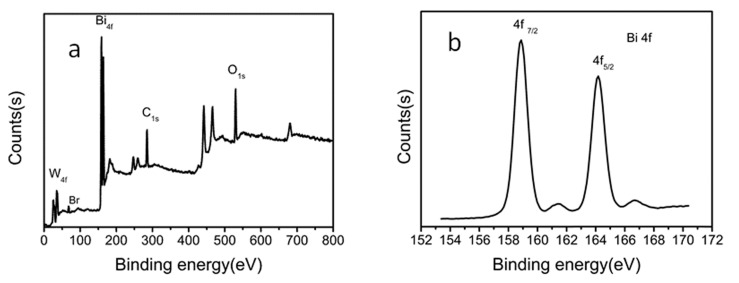

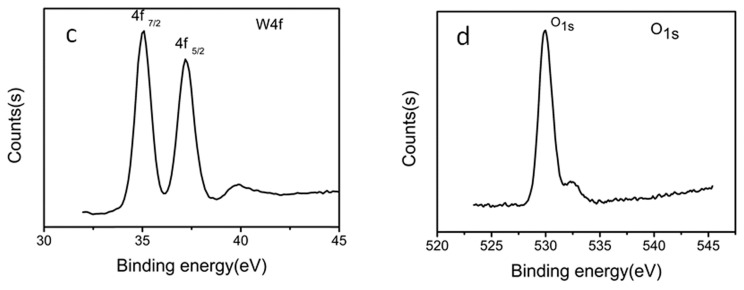
XPS spectra of the Bi_2_WO_6_ nanoplates: (**a**) the typical survey, the high-resolution spectra of (**b**) Bi_4f_; (**c**) W_4f_ and (**d**) O_1s_.

An X-ray photoelectron spectroscopy (XPS) analysis on our obtained two-dimensional thin Bi_2_WO_6_ nanoplates was performed in order to study the chemical states and surface composition of the elements existing in the Bi_2_WO_6_ thin nanoplates (Figure 2). Figure 2a is the XPS full survey spectra on Bi_2_WO_6_ thin nanoplates, where we can see the existence of Bi, W and O elements in Bi_2_WO_6_ nanoplates (C element belongs to the binder), and a tiny peak at 68.38 eV can be attributed to the presence of Br element [15] (the EDS pattern in Figure S1 also indicates the existence of Br element), which will possibly increase the adsorption and photocatalytic activity of Bi_2_WO_6_ nanoplates according to the reported literature results [15]. 

The high-resolution spectra survey of separate Bi, W and O elements were displayed respectively as follows: two peaks centered at 158.8 eV and 164.2 eV (Figure 2b) could be attributed to the relevant 4f_7/2_ and 4f_5/2_ of Bi^3+^ element, the binding energies of 35.1 eV and 37.2 eV (Figure 2c) are separately assigned to 4f_7/2_ and 4f_5/2_ from W^6+^ element and the non-symmetric O_1s_ peak located at 529.8 eV should be due to the contributions from crystal lattice oxygen and adsorbed oxygen existent in the oxides (Figure 2d) [11,16].

**Figure 3:**
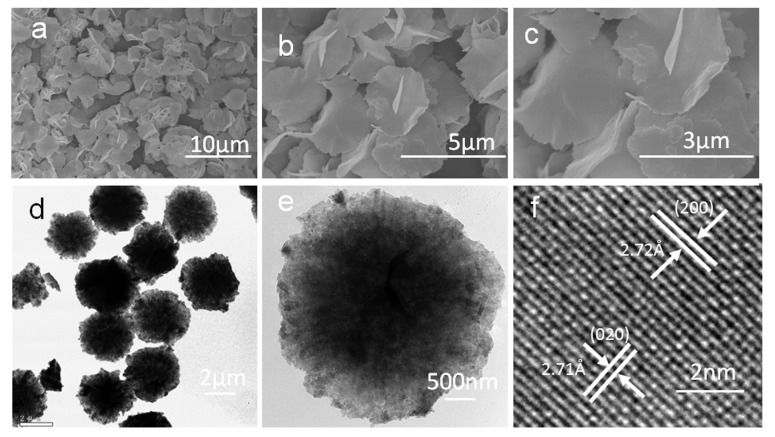
(**a**) General and (**b**,**c**) higher magnification SEM images of Bi_2_WO_6_ thin nanoplates; (**d**,**e**) TEM images of Bi_2_WO_6_ thin nanoplates; (**f**) HRTEM image taken on a certain part of Bi_2_WO_6_ nanoplate.

The formation and morphologies of two-dimensional Bi_2_WO_6_ thin nanoplates can be demonstrated by their FESEM and TEM images. Figure 3a shows a panoramic FESEM image of a representative Bi_2_WO_6_ sample made up of abundant dispersive thin nanoplates with sizes of ca. 3.0 µm. The higher FESEM magnification images in Figure 3b,c clearly reveal Bi_2_WO_6_ nanoplates displaying thin and two-dimensional microstructures, from which the thickness of the Bi_2_WO_6_ nanoplates was measured as around 60 nm.

**Figure 4:**
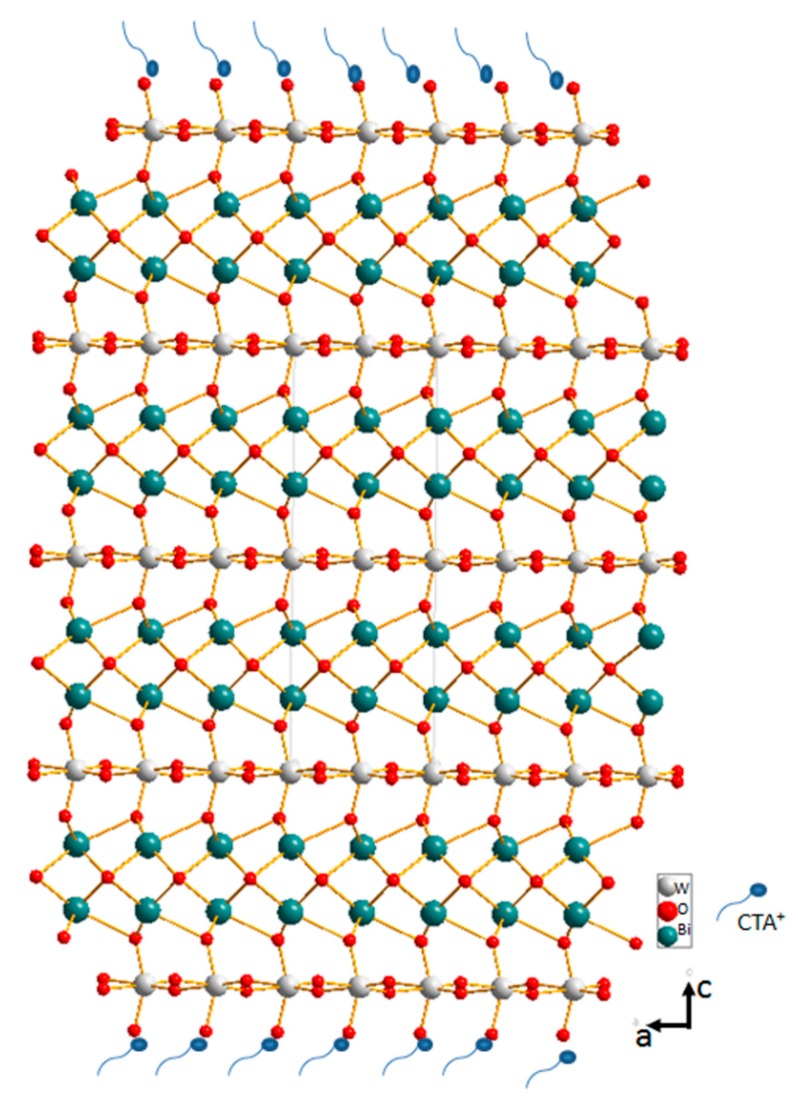
Structural model of the Bi_2_WO_6_ nanoplates.

Additionally, Panels (d) and (e) of Figure 3 show the bright-field TEM images of Bi_2_WO_6_ nanoplates, which further indicate the two-dimensional microstructures of the Bi_2_WO_6_ nanoplates. Panel (f) of Figure 3 shows the enlarged lattice-resolved HRTEM image from a certain part of a single Bi_2_WO_6_ nanoplate, where the spacing of the observed lattice plane is approximately 0.272 nm and 0.271 nm, consistent with the spacing for the (200) and (020) planes of orthorhombic Bi_2_WO_6_. This indicated that the growth orientation of two-dimensional Bi_2_WO_6_ nanoplates is preferentially along the (001) basal plane in accordance with the XRD analysis (Figure 1) and we may speculate that the two-dimensional Bi_2_WO_6_ nanoplates with more exposed (001) planes will show higher visible light-driven photocatalytic activities than Bi_2_WO_6_ microstructures with other exposed crystal planes [11,17,18].The formation mechanism of our prepared two- dimensional Bi_2_WO_6_ nanoplates in the present reaction system could be attributed to the CTAB-assisted hydrothermal fabrication method used, as CTAB surfactant is a widely used cationic ligand in colloidal chemistry and usually plays an important role as a soft template for the shape and morphology control of semiconductor compounds [15,19,20]. Here in the as-obtained two-dimensional thin Bi_2_WO_6_ nanoplates, CTAB makes a big difference in controlling the shape and exposed facets of Bi_2_WO_6_ microstructures and only three-dimensional hierarchical microspheres were obtained in the absence of CTAB while keeping other fabrication conditions unchanged (Figure S2), therefore combined with the simulated Bi_2_WO_6_ crystalline structures (numerous oxygen atoms existing in oxygen bridge bonds on the surface of (001) facets, Figure 4), CTA^+^ cations could selectively adsorb on the surfaces of (001) facets of Bi_2_WO_6_ crystal reducing their surface energy. In view of the fact that the lattice planes with higher surface energy will eliminate by faster growth than lattice planes with lower surface energy [14,21,22] and the surfaces (001) facets was probably lower than that of other facets, thus unique two-dimensional Bi_2_WO_6_ thin nanoplates with more exposed (001) facets caused by the preferential overgrowth were fabricated in this reaction solution.

### 3.2. UV-Vis DRS Analysis

**Figure 5:**
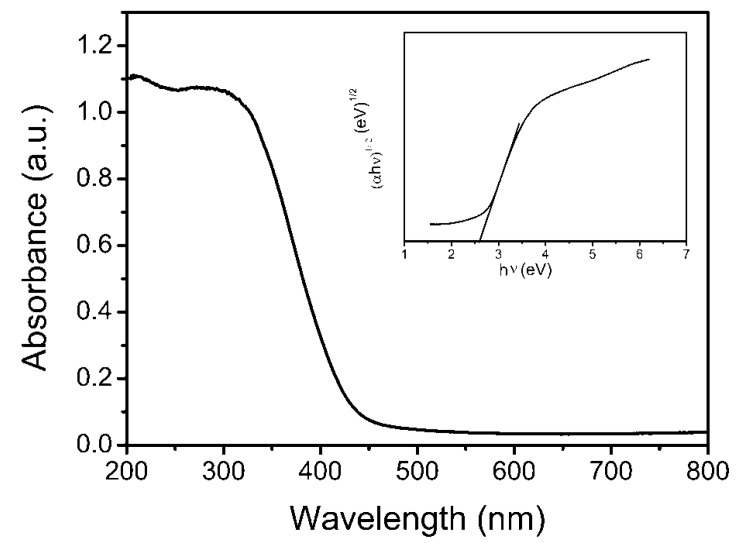
UV-vis diffuse reflectance spectra of Bi_2_WO_6_ nanoplates. The insert is the corresponding (αhν)^1/2^ versus photon energy plots.

The optical absorption property of fabricated two-dimensional thin Bi_2_WO_6_ nanoplates was investigated by the UV-visible diffuse reflectance spectroscopy technique (Figure 5), which indicated that thin two-dimensional Bi_2_WO_6_ nanoplates exhibited strong photo-absorption in visible light region with a steep absorption edge of 451 nm. We can infer that the visible light absorption was caused by intrinsic band-gap transitions but not of transitions from the impurity level [23]. As regard to a crystalline semiconductor, the optical absorption near the band edge is in step with the equation αhν = A (hν − Eg)^n^ for a direct band gap material, where α, h, ν, Eg and A are respectively the absorption coefficient, Plank constant, light frequency, band gap and a constant, while n resolved the feature of the transition in a semiconductor and the n value of two-dimensional thin Bi_2_WO_6_ nanoplates equals to 2 [24]. The optical band gap (Eg) acquired by extrapolation of the plot (the inset of Figure 5) of (αhν)^1/2^ versus hν is around 2.62 eV, close to that of the previously reported Bi_2_WO_6_ microstructures in literature (2.64 eV) [25]. The color of the obtained thin Bi_2_WO_6_ nanoplates was light-yellow, as can also be expected from the absorption spectrum. On the basis of the above UV-Vis DRS analysis results, we may infer that the as-obtained thin Bi_2_WO_6_ nanoplates should possess the capability for visible light-driven photocatalytic degradation of organic pollutants in aqueous solution in the water treatment field.

### 3.3. BET Surface Area Analysis

**Figure 6:**
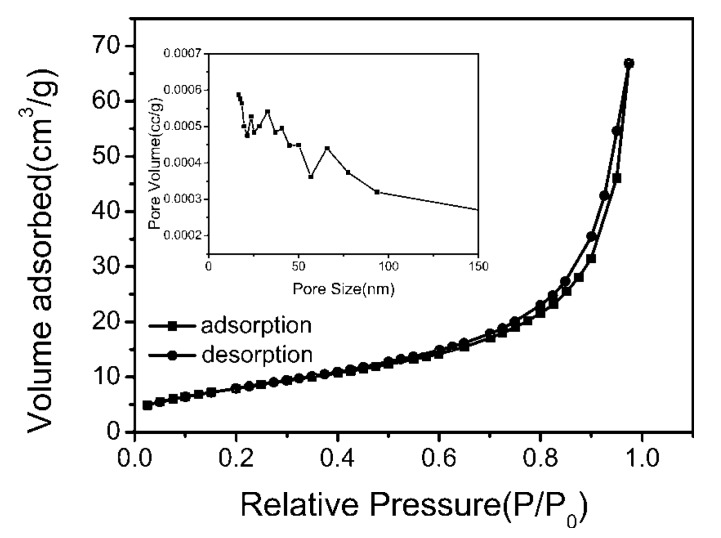
N_2_ adsorption and desorption isotherms and pore size distribution curve (insert) for Bi_2_WO_6_ nanoplates.

The Brunauer-Emmett-Teller (BET) specific surface area and the pore size distribution (PSD) of the fabricated two-dimensional thin Bi_2_WO_6_ nanoplates were studied by the N_2_ adsorption and desorption isotherms and the corresponding PSD curves, as shown in Figure 6. The adsorption—desorption isotherm for Bi_2_WO_6_ nanoplates is of type IV, implying the presence of mesopores (size of 2–50 nm) [26], while the examined hysteresis loop extends to a higher relative pressure with value of P/P_0_ around 1 indicating the presence of macropores (size larger than 50 nm) [27]. The pore size distribution curve (inset in Figure 6) of the two-dimensional thin Bi_2_WO_6_ nanoplates has main peaks at 23, 33 and 66 nm, so we can infer that mesoporous structures (23 nm–50 nm) can be assigned to tiny pores in individual thin Bi_2_WO_6_ nanoplates and macropores (larger than 66 nm) are mainly caused by aggregation of Bi_2_WO_6_ nanoplates associated with the above FESEM images shown in Figure 3. The BET surface area of two-dimensional thin Bi_2_WO_6_ nanoplates was calculated to be 29.76 m^2^/g from the N_2_ adsorption and desorption isotherms, higher than that of typical SSR-Bi_2_WO_6_ (0.6 m^2^/g) [28]. Considering the larger BET surface area may be effective for efficient visible light harvesting, the visible light-driven photocatalytic activity of Bi_2_WO_6_ two-dimensional thin Bi_2_WO_6_ nanoplates will be increased.

### 3.4. Photocatalytic Activity

It was considered that a high adsorption capability of the organic pollutants on the surface of a semiconductor photocatalyst can not only strengthen the photocatalytic activity, but also expand the practical large-scale applicability for efficiently removing hazardous pollutants in the dark or under weak light irradiation, where the photocatalyst figures as a collector to gather the pollutants in aqueous solution [29,30]. Hence semiconductor photocatalysts with enhanced adsorption capability and photocatalytic activity are desirable for high removal of organic pollutants in the water treatment field.

**Figure 7:**
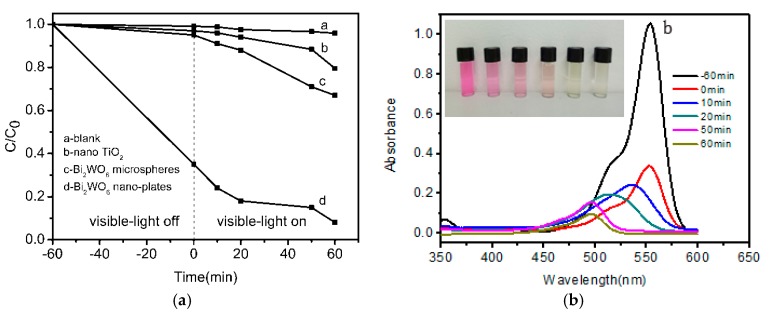
(**a**) Comparison of photocatalytic activities on degradation of RhB from Bi_2_WO_6_ nanoplates, microspheres and nano TiO_2_; (**b**) The temporal evolution of the absorption spectra of the RhB solution under visible-light irradiation in the presence of 50 mg Bi_2_WO_6_ nanoplates, insert is the color changes of the RhB aqueous solution.

Here the photocatalytic activities of the presently obtained two-dimensional thin Bi_2_WO_6_ nanoplates was evaluated by using the organic pollutant RhB as target molecule in aqueous solution under visible light irradiation (Figure 7a).

The blank test indicated that the photo-degradation efficiency was only 4% within 60 min, thus the RhB degradation is particularly slow without Bi_2_WO_6_ nanoplates under visible light irradiation. Fortunately we found that the two-dimensional thin Bi_2_WO_6_ nanoplates displayed much higher adsorption abilities and more enhanced visible light-driven photocatalytic performance than that of nano-TiO_2_ and of Bi_2_WO_6_ microspheres precipitated in the absence of CTAB in reaction solution, whereby 65% of the RhB organic pollutant molecules were adsorbed on the surface of the thin Bi_2_WO_6_ nanoplates after 60 min absolute adsorption–desorption equilibrium without any light irradiation, and after that the RhB organic dye was almost completely degraded after 60 min of visible light illumination. An influence of the initial concentration of RhB aqueous solution on the photo-degradation performance over Bi_2_WO_6_ nanoplates was observed (Figure S3), as when the initial concentration was lowered to 2 × 10^−6^ mol/L, the adsorption of RhB in the dark was more than 70% and the remaining RhB was fully degraded within only 10 min, but the degradation performance was weakened when the initial concentration of RhB was increased to 2.5 × 10^−5^ mol/L, therefore the photo-degradation performance of Bi_2_WO_6_ nanoplates differed by changing the concentration of RhB in aqueous solution. When the amount of Bi_2_WO_6_ nanoplate photocatalyst was decreased to 20 mg, both the adsorption and the photocatalytic performance were weaker than that of 50 mg Bi_2_WO_6_ (Figure S4). The enhanced visible light-driven photocatalytic performance of two-dimensional thin Bi_2_WO_6_ nanoplates can be attributed to their more exposed (001) facets [18, 31], which were beneficial for promoting charge transfer and further highlighted the photocatalytic activities of Bi_2_WO_6_ nanoplates under visible light irradiation. Figure 7b exhibits the temporal evolution of the absorption spectra of RhB aqueous solution degraded by Bi_2_WO_6_ nanoplates under visible light irradiation. It is clearly observed that the intensity of the major absorption peaks from RhB decreased gradually with extended time, and the absorption peaks disappeared almost completely when the irradiation time reached 60 min, which illustrates a nearly complete degradation of RhB, consistent with the colour changes of the suspension from initial pink to transparent (insert of Figure 7b). It is noteworthy that two-dimensional thin Bi_2_WO_6_ nanoplates with exposed (001) facets could provide more reactive sites for sunlight harvesting and the adsorption, degradation of organic dye molecules as well, thus the visible light-driven photocatalytic efficiency of Bi_2_WO_6_ nanoplates was enhanced. In order to further demonstrate the visible light-driven photocatalytic activity of Bi_2_WO_6_ nanoplates, the photo-degradation of colorless phenol as a substrate was performed [32], Figure S5 shows that the photo-degradation efficiency of phenol was 39% after 6 h, which further demonstrates the visible light-driven photocatalytic capability from Bi_2_WO_6_ nanoplates on organic pollutant aqueous solution degradation. 

## 4. Conclusions

In summary, two-dimensional Bi_2_WO_6_ thin nanoplates were prepared in the presence of CTAB using a hydrothermal route as a semiconductor photocatalyst with high adsorption and visible light-driven photocatalytic performance efficient on removal of environmental pollutants in the water treatment field. The proposed formation mechanism and visible light-driven photocatalytic activity of thin Bi_2_WO_6_ nanoplates were investigated. In view of the fact more exposed (001) facets of Bi_2_WO_6_ nanoplates with a typical two-dimensional nanoplate microstructure may lead to more detached photogenerated charge carriers, and the active sites on the catalyst surface will grow in number, therefore the visible light-driven photocatalytic activity of Bi_2_WO_6_ thin nanoplates on degradation of organic pollutants will be greatly enhanced, indicating possible application of the as-prepared two-dimensional Bi_2_WO_6_ thin nanoplates in the water treatment field.

## Supplementary Materials

The following are available online: Figure S1. EDS pattern of Bi_2_WO_6_ thin nanoplates prepared at 180°C for 20h; Figure S2. SEM images of Bi_2_WO_6_ microspheres, 180°C, 20h; Figure S3. Comparison of photocatalytic activities on degradation of different concentration of RhB from Bi_2_WO_6_ nanoplates (a) 2.5 × 10^-5^, (b) 1 × 10^-5^, (c) 2 × 10^-6^; Figure S4. Comparison of photocatalytic activities on degradation of RhB (1 × 10^-5^) from different amounts of Bi_2_WO_6_ nanoplates (a) 20mg, (b) 50mg; Figure S5. Photocatalytic activity on degradation of color-less phenol (15 mg/L) of Bi_2_WO_6_ nanoplates.
