# Supplementary material for: CTAB-Assisted Fabrication of Bi2WO6 Thin Nanoplates with High Adsorption and Enhanced Visible Light-Driven Photocatalytic Performance
Source: Molecules. 2017 May 22;22(5):859. doi: 10.3390/molecules22050859 (PMC6153929; doi:10.3390/molecules22050859)
Supplement: Supplementary file 1 [file molecules-22-00859-s001.pdf]

Supplementary Materials

CTAB-Assisted Fabrication of Bi_2WO_6 Thin Nanoplates with High Adsorption and Enhanced Visible Light–Driven Photocatalytic Performance

Yuxue Zhou, Pengfei Lv, Xiangdong Meng, Yanping Tang, Pingping Huang, Xiaobing Chen, Xiaoshuang Shen and Xianghua Zeng

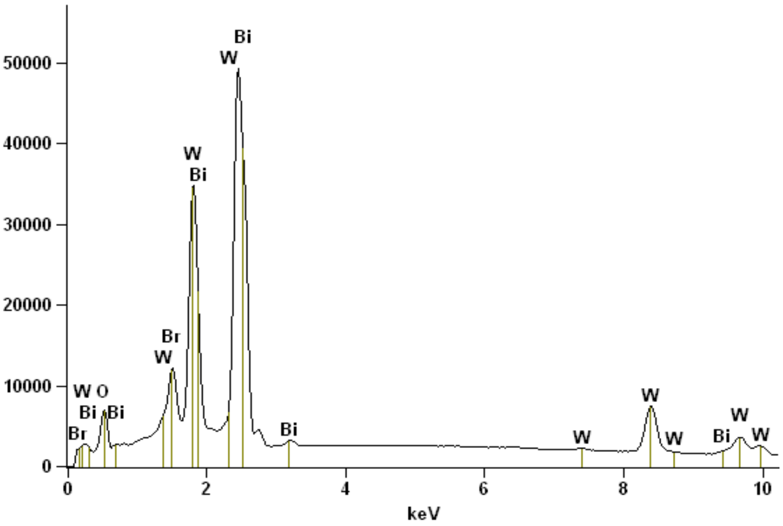

Figure S1. EDS pattern of Bi_2WO_6 thin nanoplates prepared at 180°C for 20h.

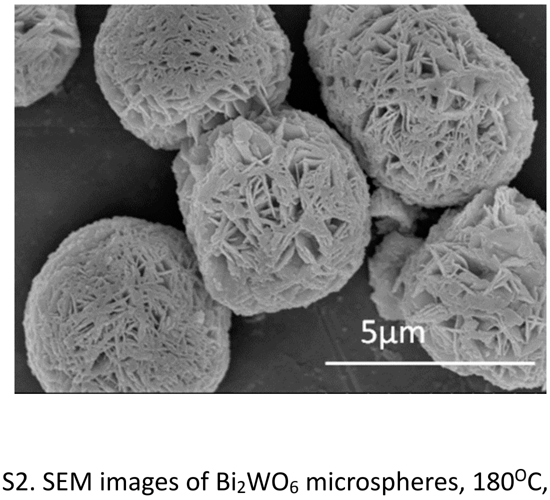

Figure S2. SEM images of Bi_2WO_6 microspheres, 180°C, 20h.

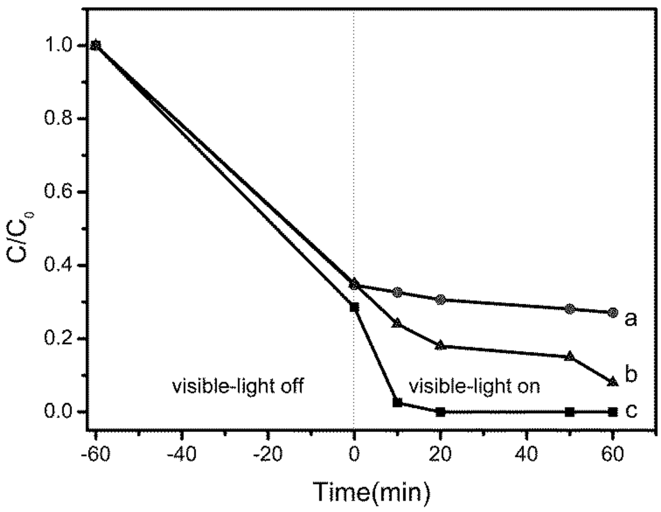

Figure S3. Comparison of photocatalytic activities on degradation of different concentration of RhB from Bi_2WO_6 nanoplates (a) 2.5×10^{-5}, (b) 1×10^{-5}, (c) 2×10^{-6}.

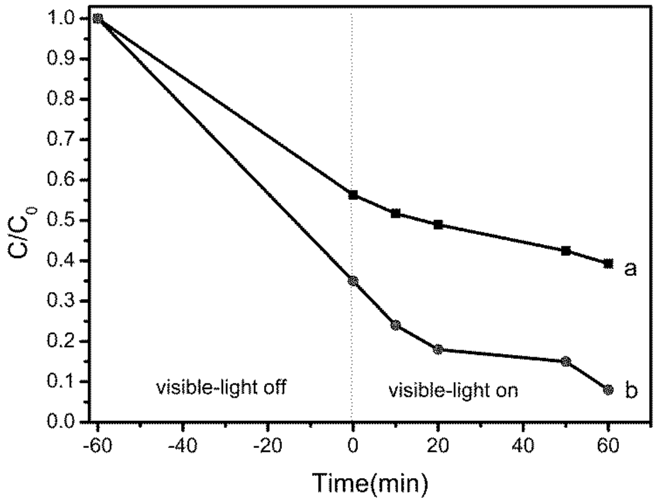

Figure S4. Comparison of photocatalytic activities on degradation of RhB (1×10^{-5}) from different amounts of Bi_2WO_6 nanoplates (a) 20mg, (b) 50mg.

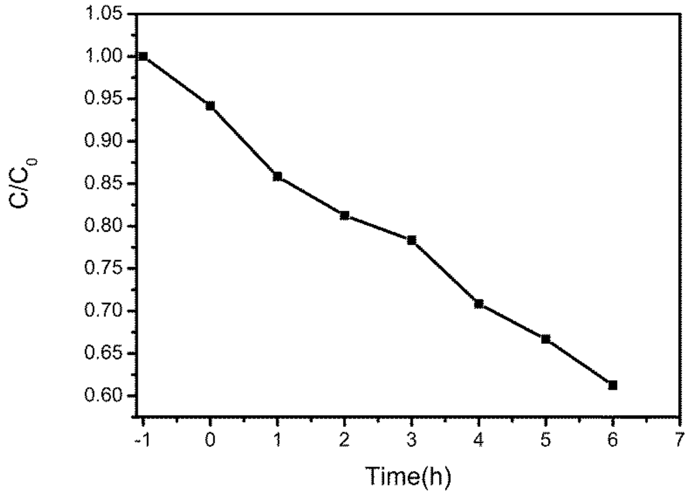

Figure S5. Photocatalytic activity on degradation of color-less phenol (15mg/L) of Bi_2WO_6 nanoplates.
